# Myosin X Interaction with KIF13B, a Crucial Pathway for Netrin-1-Induced Axonal Development

**DOI:** 10.1523/JNEUROSCI.0929-20.2020

**Published:** 2020-11-25

**Authors:** Hua-Li Yu, Yun Peng, Yang Zhao, Yong-Sheng Lan, Bo Wang, Lu Zhao, Dong Sun, Jin-Xiu Pan, Zhao-Qi Dong, Lin Mei, Yu-Qiang Ding, Xiao-Juan Zhu, Wen-Cheng Xiong

**Affiliations:** ^1^Department of Neurosciences, School of Medicine, Case Western Reserve University, Cleveland, Ohio 44106; ^2^Department of Neuroscience and Regenerative Medicine, Medical College of Georgia, Augusta University, Augusta, Georgia 30912; ^3^Institute of Cytology and Genetics, Northeast Normal University, Changchun 130024, People's Republic of China; ^4^Institute of Brain Sciences, Fudan University, Shanghai 200031, People's Republic of China

**Keywords:** axon, KIF13B, Myo X, Netrin-1, KIF13B, axon initiation, axon branching

## Abstract

Myosin X (Myo X) transports cargos to the tips of filopodia for cell adhesion, migration, and neuronal axon guidance. Deleted in Colorectal Cancer (DCC) is one of the Myo X cargos that is essential for Netrin-1-regulated axon pathfinding. The function of Myo X in axon development *in vivo* and the underlying mechanisms remain elusive. Here, we provide evidence for the role of Myo X in Netrin-1-DCC-regulated axon development in developing mouse neocortex. The knockout (KO) or knockdown (KD) of Myo X in cortical neurons of embryonic mouse brain impairs axon initiation and contralateral branching/targeting. Similar axon deficits are detected in Netrin-1-KO or DCC-KD cortical neurons. Further proteomic analysis of Myo X binding proteins identifies KIF13B (a kinesin family motor protein). The Myo X interaction with KIF13B is induced by Netrin-1. Netrin-1 promotes anterograde transportation of Myo X into axons in a KIF13B-dependent manner. KIF13B-KD cortical neurons exhibit similar axon deficits. Together, these results reveal Myo X-KIF13B as a critical pathway for Netrin-1-promoted axon initiation and branching/targeting.

**SIGNIFICANCE STATEMENT** Netrin-1 increases Myosin X (Myo X) interaction with KIF13B, and thus promotes axonal delivery of Myo X and axon initiation and contralateral branching in developing cerebral neurons, revealing unrecognized functions and mechanisms underlying Netrin-1 regulation of axon development.

## Introduction

Neurons are highly polarized cells typically with a single axon and multiple dendrites. Axon development is crucial for the establishment of neuronal connections, especially for the connection between different brain regions. Axon development includes the following three main steps: (1) axon specification/initiation during neuronal polarization; (2) axon growth and guidance; and (3) axon branching and presynaptic differentiation. During early development of mammalian cortex, the migrating neurons in the intermediate zone (IZ) transit from multipolar to bipolar, with a leading process toward the pia and a trailing process toward the ventricle. Once localized into the cortical plate (CP), the leading processes will develop into highly branched dendrites and the trailing processes will become long axons projecting to target regions for further bifurcation (Barnes and Polleux, 2009; Yogev and Shen, 2017). Eventually, axons connect with target neurons to form synapses that are crucial for neurotransmission.

Axon development is regulated by intrinsic factors in neurons as well as by microenvironmental factors or extracellular guidance cues. Netrin-1 belongs to the netrin family of extracellular guidance cues, which is crucial for axon pathfinding (Colamarino and Tessier-Lavigne, 1995; Braisted et al., 2000). Netrin-1 exerts attractive and repulsive effects through two families of receptors, Deleted in Colorectal Cancer (DCC) and UNC-5, respectively (Hedgecock et al., 1990; Ackerman et al., 1997; Leonardo et al., 1997; Culotti and Merz, 1998; Hong et al., 1999; Keleman and Dickson, 2001). Various signaling cascades are involved in Netrin-1-DCC-induced neurite outgrowth and/or growth cone guidance in cultured neurons, which include Rho family GTPases (Li et al., 2002; Shekarabi and Kennedy, 2002), phospholipase C (Xie et al., 2006), phosphoinositol 3-kinase (PI3K; Ming et al., 1999), extracellular signal-regulated protein kinase (Campbell and Holt, 2003; Forcet et al., 2002; Ming et al., 2002), FAK (Li et al., 2004; Liu et al., 2004; Ren et al., 2004), PITPalpha (Xie et al., 2005), and Myosin X (Myo X; Zhu et al., 2007). Recent studies using Netrin-1 CKO mice suggest that the ventricular zone-derived Netrin-1 contributes to commissural axon projection by bounding to commissural axons near the pial surface (Dominici et al., 2017). This finding makes the function of locally produced Netrin-1 in commissural axon development more prominent. However, how Netrin-1 regulates cortical neuronal axon development or projection remains to be determined.

Myo X, an unconventional actin-based motor protein, is primarily localized at the tips of filopodia or the edges of lamellipodia and membrane ruffles (Berg et al., 2000; Berg and Cheney, 2002; Tokuo and Ikebe, 2004; Zhang et al., 2004). It undergoes forward and rearward movements within filopodia and promotes filopodia formation, elongation, and sensing, possibly by transporting actin binding proteins and cell adhesion receptors to the leading edge of the cells (Berg and Cheney, 2002; Tokuo and Ikebe, 2004; Zhang et al., 2004; Tokuo et al., 2007; Zhu et al., 2007). Myo X has a unique protein structure feature, containing a motor domain at its N terminus, three calmodulin-binding IQ motifs, 3 PH (pleckstrin homology) domains, one myosin tail homology (MyTH) domain, and one band 4.1-ezrin-radixin-meosin (FERM) domain (Berg et al., 2000; Yonezawa et al., 2000). Via these domains, Myo X not only binds to F-actin filaments, but also interacts with phosphoinositol lipids, microtubules, and transmembrane receptors (e.g., integrins and DCC family receptors; Cox et al., 2002a; Tokuo and Ikebe, 2004; Weber et al., 2004; Zhang et al., 2004; Zhu et al., 2007; Plantard et al., 2010). In cultured neurons, Myo X is gradually accumulated in nascent axons, where it regulates axon outgrowth (Yu et al., 2012). In chicken embryos, expression of motorless Myo X impairs axon growth and commissural axon midline crossing (Zhu et al., 2007). Notice that Myo X is critical for transporting DCC to the dynamic actin-based membrane protrusions (Zhu et al., 2007), and, on the other hand, the motor activity and distribution of Myo X are also reciprocally regulated by DCC and neogenin (Liu et al., 2012). Whereas these observations support the view for Netrin-1–DCC–Myo X pathway to be critical for axon development, whether and how they regulate axon development *in vivo* remain to be further elucidated.

Here, we present evidence for Myo X interaction with KIF13B to be crucial for Netrin-1-induced axon initiation and branching/targeting in developing mouse cortical brain. Myo X interacts with KIF13B (also called GAKIN), a kinesin family member that is essential for delivery of phosphatidylinositol (3,4,5)-trisphosphate [PI(3,4,5)P3] to axons and axon outgrowth (Horiguchi et al., 2006; Yoshimura et al., 2010). Netrin-1 increases Myo X interaction with KIF13B, thus promoting anterograde transport and axonal targeting of Myo X in neurons. Such Netrin-1 function requires Myo X interaction with DCC and KIF13B, as well as PI3K activity. Additionally, as Netrin-1 and DCC, both Myo X and KIF13B are required for axon initiation and contralateral branching/targeting in developing cerebral cortex. Together, these results suggest that by promoting KIF13B-mediated axonal transport of Myo X, Netrin-1 plays critical roles in inducing axonal initiation and enhancing axonal contralateral branching, revealing unrecognized functions and mechanisms underlying the Netrin-1 signaling pathway in axon development.

## Materials and Methods

### 

#### 

##### Animals

Myo X^f/f^ mice were generated as previously described (Wang et al., 2019), and Netrin-1 floxed (NTN1^f/f^) mice were kindly provided by Yu-Qiang Ding (Fudan University, Shanghai, China). All the mouse lines indicated above were maintained on a C57BL/6 background for more than six generations. Timed pregnant female mice were obtained by crossing with male mice overnight, and the noon of the following day was designated as embryonic day 0.5 (E0.5) if the vaginal plug was detected.

##### Reagents

For immunostaining analysis, the following primary antibodies were used: mouse monoclonal anti-Tau-1 (catalog #05–838; 1:1000), mouse monoclonal anti-MAP2 (catalog #05–346; 1:500), rat monoclonal anti-tubulin (catalog #MAB1864; 1:500) from Millipore; chicken polyclonal anti-GFP (catalog #ab13970; 1:1000) and mouse monoclonal anti-c-Myc (catalog #ab32; 1:200) from Abcam. For immunoblotting analysis, the following primary antibodies were used: goat polyclonal anti-DCC (catalog #sc-6535; 1:200) from Santa Cruz Biotechnology; rabbit polyclonal anti-KIF13B (catalog #PA540807;1:500) from Thermo Fisher Scientific; mouse monoclonal anti-α-tubulin (catalog #T5168; 1:4000) and mouse monoclonal anti-GFP (catalog #11814460001; 1:2000) from Sigma-Aldrich; rabbit polyclonal anti-Myc (catalog #ab9106; 1:1000) from Abcam; rabbit polyclonal anti-MyoX was prepared as previously described (Zhu et al., 2007); the polyclonal anti-KIF13B antibody was provided by Hiroaki Miki (Osaka University, Osaka, Japan). For immunoprecipitation assay, rabbit polyclonal anti-GFP (catalog #A11122) was purchased from Thermo Fisher Scientific. Alexa Fluor 488-, 555-, and 647-coupled secondary antibodies against mouse, rat, chicken, or goat and HRP-conjugated secondary antibodies against mouse or rabbit were purchased from Jackson ImmunoResearch. Alexa Fluor 350-phalloidin (catalog #A22281) and lipofectamine 2000 (catalog #11 668–019) were obtained from Thermo Fisher Scientific. Wortmannin was from Millipore.

##### Expression vectors

The cDNA of mouse *Myo X* was subcloned into mammalian expression vector (pEGFP-C1) fused with GFP at the N terminus, as described previously (Zhu et al., 2007). GFP-MyoXΔPH and GFP-MyoXΔFERM were generated with a Q5 Site-Directed Mutagenesis Kit (catalog #E0554S, New England Biolabs), where the amino acids 1212–1253 and 1799–2058 were deleted, respectively. Myo X-Head, Myo XΔMotor (headless Myo X, hMyo X), Myo X MyTH4-FERM, and MyoX FERM were amplified and subcloned into pEGFP-C1. GFP-hMyoXΔPH2 and GFP-hMyoXΔPH3 were generated with the Q5 Site-Directed Mutagenesis Kit, where the amino acids 1215–1316 and 1381–1506, respectively, were deleted. Also, GFP-hMyoX (KK1225/6AA) were generated with the Q5 Site-Directed Mutagenesis Kit. The cDNA of human KIF13B was subcloned into mammalian expression vector (pRK5) fused with a Myc tag at the N terminus. KIF13B^1-557^, KIF13B^1-1531^, KIF13B^558-1826^, KIF13B^990-1826^, and KIF13B^1532-1826^ were amplified by PCR and subcloned into pRK5 through corresponding restriction enzymes or exonuclease III. The expression vectors of all glutathione *S*-transferase (GST) fusion proteins was constructed by ligation into pGEX-6p-1. mCherry-Myo X was kindly provided by Staffan Strömblad (Karolinska Institutet, Huddinge, Sweden).

The miRNA expression vectors for Myo X and DCC were generated by the BLOCK-iT Lentiviral miR RNA Expression System (Thermo Fisher Scientific) according to the manufacturer instruction as previously described (Zhu et al., 2007; Liu et al., 2012). The shRNA expression vectors for mouse KIF13B were generated using pll3.7 lentiviral vector, and the target sequences for KIF13B shRNAs are as follows: 5′-GCAGATAACTATGACGAAACC-3′ (KIF13B shRNA-1); and 5′-GGATTTAGCTGGCAGTGAACG-3′ (KIF13B shRNA-2). Netrin-1 shRNA was constructed into pll3.7 lentiviral vector as previously reported, and the target sequence was 5′-GGGTGCCCTTCCAGTTCTA-3′ (Chen et al., 2017). DCC shRNA was constructed into pll3.7 lentiviral vector as previously reported, and the target sequence was 5′-CTTGGAGGAAGGAGAGACA-3′ (Zhang et al., 2018). In addition, we also generated the RFP-Scramble shRNA and RFP-KIF13B shRNA expression vectors by replacing GFP with RFP in the pll3.7 plasmids. The authenticity of all constructs was verified by DNA sequence.

##### Cell cultures and transfections

Primary cortical neurons were cultured as described previously (Zhu et al., 2007). In brief, embryos (E17) were removed from anesthetized pregnant mice. Cerebral cortices were separated and chopped into small pieces. After incubation in 0.125% Trypsin plus with 0.05% DNase in HBSS at 37°C for 20 min, cells were triturated with a fire-polished glass Pasteur pipette and filtered with a 40 μm filter. Dissociated cells were suspended in DMEM with 10% FBS and plated on poly-d-lysine-coated dishes or glass coverslips at 37°C in a 5% CO_2_ atmosphere. Four hours later, the medium was changed into Neurobasal medium with 2% B27 supplement and 2 mm Glutamax. For transfection, neurons were electroporated immediately after dissociation using the Mouse Neuron Nucleofector Kit (Amaxa). In brief, 3 × 10^6^ neurons were resuspended in 100 µl of Nucleofectamine solution containing 3 µg of plasmid and electroporated with Program O-003 of Nucleofector.

NLT cells and HEK293 cells were grown in DMEM supplemented with 10% FBS and 100 U/ml penicillin-streptomycin. For imaging experiments, 50−70% confluent NLT cells in 12-well plate were transfected with 1.6 μg of indicated plasmids using 3 μl of lipofectamine in DMEM without FBS and antibiotics. For Western blot and coimmunoprecipitation, HEK293 cells were transfected using polyetherimide (PEI). Stable HEK293 cell line expressing human Netrin-1 was used as described previously (Ren et al., 2004; Xie et al., 2005; Zhu et al., 2007). The working concentration of Netrin-1 was 500 ng/ml.

##### *In utero* electroporation

The *in utero* electroporation (IUE) was conducted as described previously with some modifications (Yu et al., 2012). Briefly, plasmids were microinjected into the lateral cerebral ventricle of E14.5 or E15.5 mouse embryos through the uterine wall. Then, a 35 V square-wave pulse was delivered across the head for five times through and ECM-830 Electroporation System (BTX). Embryos were then allowed to develop to E18.5, postnatal day 7 (P7) or P14. The transfected brains were then fixed with 4% PFA/PBS overnight at 4°C. The brains were sectioned with a freezing microtome at ∼50 µm.

##### Immunostaining analysis

Cells were fixed in 4% PFA for 10 min at room temperature, permeabilized with 0.1% Triton X-100 for 8 min, and blocked in 2% bovine serum albumin for 1 h in 0.01 m PBS, pH 7.4. Subsequently, cells were incubated with primary antibodies diluted in the blocking solution for 2 h and washed three times with PBS. And they were incubated with appropriate fluorochrome-conjugated secondary antibodies for 1 h and washed three times.

##### Live cell time-lapse imaging and kymography analyses

Transfected neurons were grown on Lab-Tek II Chambered cover glass (Thermo Fisher Scientific) in DMEM supplemented with 10% FBS and antibiotics. For visualizing GFP-Myo X movement, the Lab-Tek II Chambered Coverglass were then fitted into a temperature-controlled chamber on the microscope stage of LSM 710 confocal laser-scanning microscopy (Carl Zeiss) for observation at 37°C in a 5% CO_2_ atmosphere. The time-lapse intervals were 5 s, and neurons were imaged over periods of 10 min. Imagines were acquired with a 63×/1.4 numerical aperture (NA) objective at a resolution of 1024 × 1024 pixels. The software ImageJ (FIJI) was used for tracking analysis and kymographic analysis. In brief, the traveling path and velocity of GFP-Myo X puncta were recorded with the “Manual Tracking” plugin by clicking on the GFP-Myo X puncta on the temporal stacks. For kymographic analysis, a segmented line was used to draw a region of interest (ROI) and then the “KymographBuilder” plugin was used to produce kymographs for the selected segments.

##### Fluorescence recovery after photobleaching

The experiments were performed using the LSM 710 confocal laser-scanning microscopy. Images were acquired with an 63×/1.4 N.A. objective at a resolution of 1024 × 1024 pixels. A region at the proximal axon was bleached with high laser power, and fluorescence recovery was observed for a period of 10 min. For fluorescence recovery after photobleaching (FRAP) analysis, the mean intensity of the bleached area was normalized with the initial fluorescence intensity before bleaching.

##### Mass spectrum analysis

Cultured cortical neurons transfected with GFP-hMyoX expression plasmids were lysed 48 h later with lysis buffer [20 mm Tris, pH 8.0, 10 mm NaCl, 1 mm EDTA, 0.5% NP-40, 1 mm NaF, 1 mm Na_3_VO_4_, protease inhibitor cocktail (Roche), 1 mm PMSF]. GFP-hMyoX and its binding partners were immunoprecipitated with anti-IgG (control) or anti-GFP antibodies, respectively. The immunoprecipitates were resolved by 8% SDS-PAGE gel and subjected to silver staining. All unique bands in anti-GFP group at molecular weight from ∼50–250 kDa on the gel were isolated and subjected to liquid chromatography–tandem mass spectrometry (LC-MS/MS) as previously reported (Ma et al., 2019; Applied Protein Technology). Briefly, gel pieces were destained, rehydrated, and tryptic digested with 10 ng/μl trypsin resuspended in 50 mm NH_4_HCO_3_. The tryptic peptides were dissolved in 0.1% formic acid (solvent A), directly loaded onto a reversed-phase analytical column. The gradient was composed of an increase from 6% to 23% solvent B (0.1% formic acid in 98% acetonitrile). The peptides were subjected to nanospray ionization (NSI) source followed by MS/MS in Q Exactive Plus (Thermo Fisher Scientific) coupled online to the ultra-performance LC. The resulting MS/MS data were processed using Proteome Discoverer 1.3. Tandem mass spectra were searched against UniProt database. Trypsin was specified as a cleavage enzyme allowing up to two missing cleavages. Mass error was set to 10 ppm for precursor ions and 0.02 Da for fragment ions. Carbamidomethyl on Cys were specified as fixed modification and oxidation on Met and acetylation modification were specified as variable modifications. Peptide confidence was set at high, and peptide ion score was set >20.

##### Protein–protein interaction assays

Immunoprecipitation was conducted as previously described (Ren et al., 2001). Cell lysates (1 mg of protein) were incubated at 4°C for 6 h with the indicated antibodies (1–2 μg) in a final volume of 1 ml modified RIPA lysis buffer with protease inhibitors. After the addition of protein A-G-agarose beads, each reaction was incubated at 4°C for 1 h. The immunoprecipitate complexes were collected by centrifugation and washed three times with washing buffer (20 mm Tris-HCl, 10 mm NaCl, 1 mm EDTA, 0.5% NP-40). Immune complexes were resolved by SDS-PAGE and subjected to immunoblotting. GST pulldown assay was conducted as described previously (Ren et al., 2001). Transiently transfected HEK293 cells were lysed in the modified RIPA buffer (50 mm Tris-HCl, pH 7.4, 150 mm sodium chloride, 1% NP-40, 0.25% sodium-deoxycholate, and proteinase inhibitors). Cell lysates were precleared with GST immobilized on glutathione–Sepharose 4B (GE Healthcare) and then incubated with the indicated GST fusion proteins (2–5 μg) immobilized on glutathione–Sepharose beads at 4°C overnight with constant rocking. The beads were washed three times with modified RIPA buffer, and bound proteins were resolved by SDS-PAGE and subjected to immunoblotting.

##### Imaging quantification and statistical analyses

Immunostaining sections and cells were observed under a Zeiss LSM 710 confocal microscope with ZEN 2012 software, and only the brightness, contrast, and color balance were optimized after imaging. The software ImageJ was used to measure fluorescence intensity in all fixed images. The software ImageJ was used to measure fluorescence intensity in all fixed images. In brief, the RGB images were converted into 8-bit grayscale images and inverted to negative images for analysis. After being converted to uncalibrated optical density value, the area of axons, soma, and dendrites was selected with ROI tools and calculated. GFP-Myo X intensity in axons or dendrites was normalized by that in its soma regions. Statistical analyses were performed using either unpaired two-tailed Student's *t* test or one-way ANOVA followed by a protected least significant difference Fisher's *post hoc* test for multiple comparisons. Statistical evaluations were performed with the software GraphPad Prism version 5.0. The data are presented as the mean ± SEM. *p* values <0.05 were considered significant.

## Results

### Myo X regulating axonal initiation and contralateral branching/targeting in developing cerebral cortex

**Figure 1.**
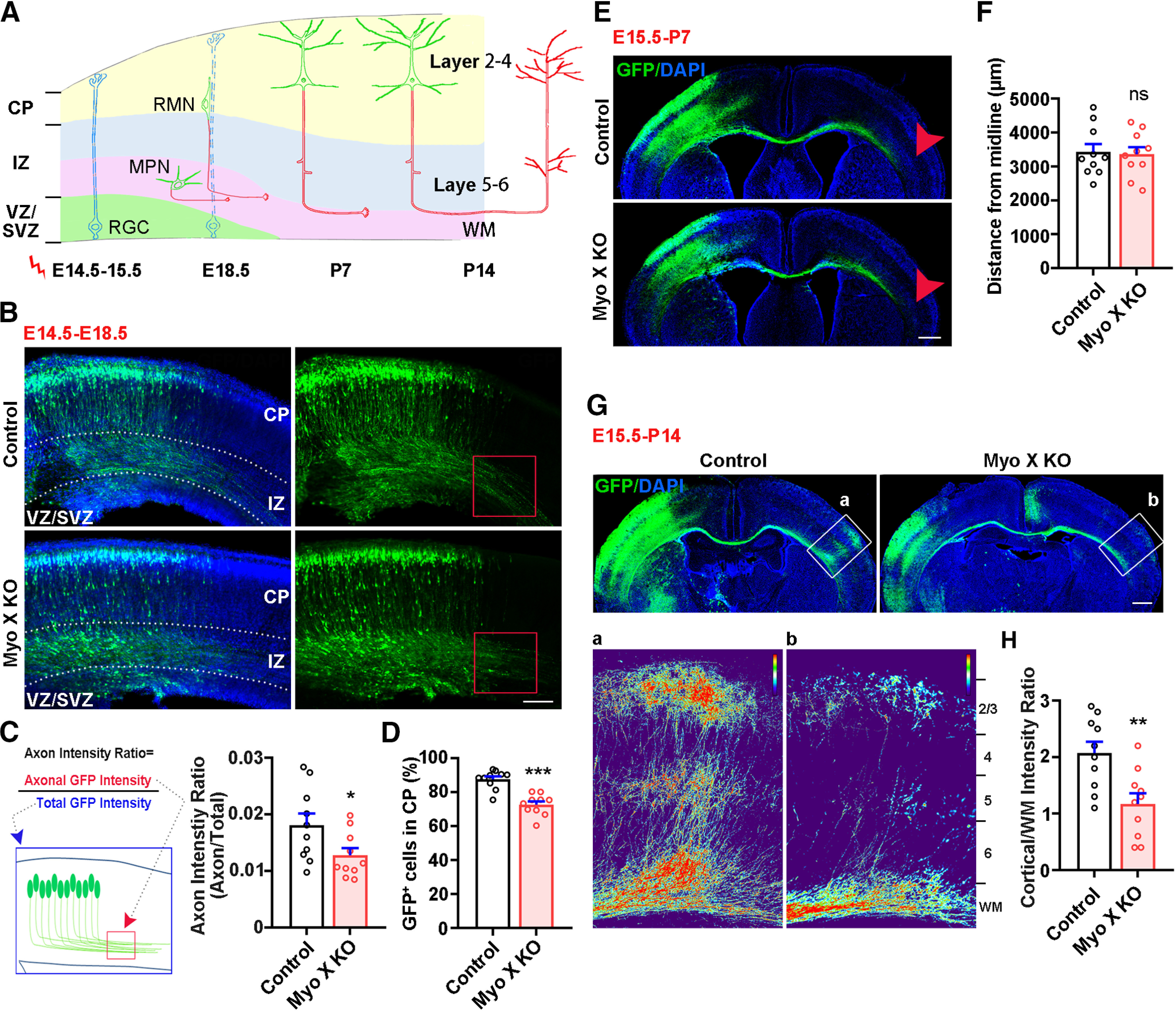
Defective axon initiation and contralateral branching in Myo X-KO cortical neurons. ***A***, Schematic diagram of IUE assay for axon development in neocortex. ***B–D***, E18.5 cerebral cortex that were electroporated with Cre-GFP plasmids into Myo X^f/f^ embryos or WT embryos at E14.5. ***B***, Representative images. Scale bar, 100 μm. ***C***, Quantification of axon initiation by using the axon intensity ratio, which is defined as axonal GFP intensity (marked by a red square) over the total GFP intensity (marked as a blue square), as illustrated in the schematic diagram. Student's *t* test, *p* = 0.0379. ***D***, Quantification of GFP-positive cells in CP. Student's *t* test, *p* < 0.0001. ***E***, ***F***, P7 cerebral cortex that were electroporated with GFP (Control) or Cre-GFP (Myo X-KO) plasmids into Myo X^f/f^ embryos at E15.5. ***E***, Representative images. Scale bar, 500 μm. ***F***, Quantifications of axon elongation. Student's *t* test, *p* = 0.84. ***G***, ***H***, P14 cerebral cortices that were electroporated with GFP (Control) or Cre-GFP (Myo X-KO) plasmids into Myo X^f/f^ embryos at E15.5. ***G***, Representative images are shown. Scale bar, 500 μm. ***H***, Quantifications of axon intensity in the contralateral cortex (as indicated by a and b in ***G***). Student's *t* test, *p* = 0.0044. Data are presented as the mean ± SEM. The numbers of brain sections scored are from three different brains for each group and indicated on the graphs. ns, No significant difference; *p < 0.05; **p < 0.01; ***p < 0.001.

To investigate the function of Myo X in axon development *in vivo*, we used Cre-LoxP recombination technology in combination with IUE to delete Myo X in portions of projection neurons in developing neocortex. Specifically, we examined the axon development of upper layer (L2–L4) neurons that project within the cortex, mostly via the corpus callosum (CC), and have birthdays at E14.5 to E15.5 (Kwan et al., 2012). IUE was thus performed at these time points, and the three key processes of axon development, including axon initiation, growth, and branching/targeting, were evaluated as illustrated in Figure 1*A*. To determine the function of Myo X in axon initiation, Cre-GFP was electroporated into the neocortex of Myo X^f/f^ and wild-type (WT) embryos at E14.5, and their axon intensity ratio (Namba et al., 2014) was analyzed at E18.5, a critical time window for axon initiation (Namba et al., 2015). As shown in Figure 1, *B* and *C*, Myo X knockout (KO) resulted in a reduction in the axon intensity ratio in the brain, suggesting a role of Myo X in this event. In addition, neuronal migration appeared to be impaired (Fig. 1*B*,*C*), as reported previously using RNA interference technology (Lai et al., 2015). We then evaluated axonal growth and midline crossing in control and MyoX-KO cortical neurons. MyoX-KO cortical neurons were achieved by IUE of Cre-GFP or GFP (as a control) into the neocortex of Myo X^f/f^ embryos (at E15.5; Wang et al., 2019); and the electroporated brain samples were examined at P7, a critical time window for cortical neuronal axon growth and midline crossing (Tagawa and Hirano, 2012; Fenlon and Richards, 2015). To our surprise, axons of Myo X-KO (Cre-GFP^+^) neurons crossed the midline, and their lengths were comparable to those of control axons (Fig. 1*E*,*F*), suggesting little role, if there is any, for Myo X to play in axon growth or middling crossing. We finally examined axon contralateral branching/targeting in P14 control and Myo X-KO cortical brains. As shown in Figure 1, *G* and *H*, axon branching/targeting to the contralateral cortex was detected in control neurons; but, this process was largely impaired in Myo X-KO neurons. These results suggest necessity of Myo X in promoting axon branching/targeting. Together, these results reveal unrecognized roles of Myo X in axon initiation and contralateral branching/targeting in developing cerebral cortex.

### Netrin-1 promoting Myo X-regulated axonal initiation and branching

**Figure 2.**
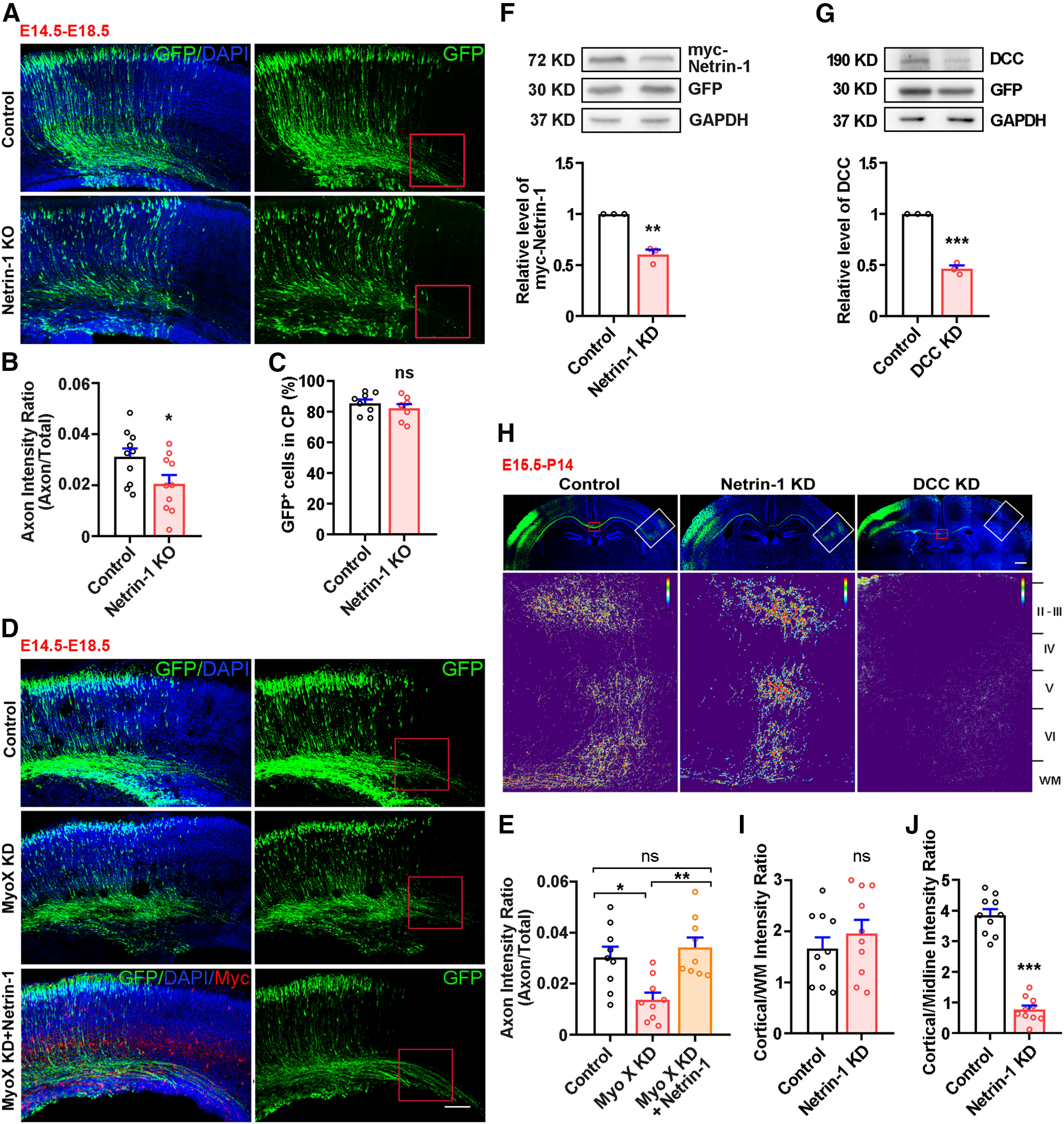
Netrin-1 rescue of axon initiation defect caused by Myo X deficiency. ***A–C***, E18.5 cerebral cortices that were electroporated with GFP (Control) or Cre-GFP (Netrin-1-KO) plasmids into NTN1^f/f^ embryos at E14.5. ***A***, Representative images. Scale bar, 100 μm. ***B***, Quantification of axon intensity ratio. Student's *t* test, *p* = 0.0418. ***C***, Quantification of GFP^+^ cells in CP. Student's *t* test, *p* = 0.39. ***D***, Representative images of E18.5 cerebral cortex electroporated with control miRNA (Control), Myo X miRNA (Myo X KD), or Myo X miRNA together with Myc-Netrin-1 plasmids (Myo X KD + Netrin-1) at E14.5. Scale bar, 100 μm. ***E***, Quantification of axon intensity ratio. One-way ANOVA: *p* = 0.0303 for the MyoX KD group; *p* = 0.74 for Myo X KD + Netrin-1 group. ***F***, ***G***, Immunoblotting analysis of Netrin-1-KD and DCC-KD efficiency. ***F***, HEK293T cells were transfected with a scrambled or Netrin-1 shRNA together with Myc-Netrin-1 plasmids for 48 h. Cell lysates were extracted for Myc immunoblotting. Student's *t* test was used for the statistical analysis of Netrin-1-KD efficiency, *p* = 0.0012. ***G***, NLT cells were transfected with a control or DCC miRNA for 48 h. Cell lysates were extracted for DCC immunoblotting. Student's *t* test was used for the statistical analysis of Netrin-1-KD efficiency, *p* < 0.0001. ***H***, Representative images of P14 cerebral cortex electroporated with Control miRNA (Control), Netrin-1 shRNA (Netrin-1 KD), and DCC miRNA (DCC KD) at E15.5. Scale bar, 500 μm. ***I***, Quantification of axon contralateral branching. Student's *t* test, *p* = 0.39. ***J***, Quantification of axon contralateral branching by using the cortical/midline intensity ratio, which is defined as axonal GFP intensity (marked by a white rectangle) over the midline GFP intensity (marked as a red rectangle), as illustrated in the schematic diagram. Student's *t* test, *p* < 0.0001. Data are presented as the mean ± SEM. The numbers of brain sections scored are from three different brains for each group and indicated on the graphs. ns, No significant difference; *p < 0.05; **p < 0.0; ***p < 0.001.

Given the specific orientation of the trailing processes or nascent axons, we speculate that extracellular signals from ventricular zone (VZ) and subventricular zone (SVZ) modulate intrinsic signaling (e.g., Myo X) for axon initiation and development, and Netrin-1, an upstream Myo X regulator (Zhu et al., 2007), may be involved in axon initiation and branching. To test this speculation, we examined the function of Netrin-1 in axon development by IUE of Cre-GFP into Netrin-1 floxed (NTN1^f/f^) embryos at E14.5. The electroporated brain samples were examined at E18.5 to evaluate the neuronal axon intensity ratio. Indeed, a reduction in the axon intensity ratio of cortical neurons was detected in Netrin-1-KO embryos (Fig. 2*A*,*B*), revealing a similar role of Netrin-1 as that of Myo X in axon initiation. Neuronal migration was not affected by Netrin-1 KO (Fig. 2*A*,*C*). We next asked whether Netrin-1-regulated axon initiation depends on Myo X. To this end, plasmids encoding Myc-Netrin-1 and Myo X miRNA were coelectroporated into the E14.5 embryos (Fig. 2*D*). Netrin-1 ectopic expression restored the axon intensity ratio in Myo X-KD neurons (Fig. 2*D*,*E*), supporting the view for Netrin-1-Myo X pathway in promoting axon initiation.

We then asked whether the Netrin-1–DCC pathway plays a role in axon branching/targeting similar to that of Myo X. Netrin-1 or DCC expression in E15.5 cortical neurons were suppressed by IUE of their shRNAs, respectively. The knock-down efficiency of Netrin-1 or DCC shRNA was examined by Western blot (Fig. 2*F*,*G*). Their axons at the age of P14 were examined. Netrin-1 KD in E15.5 cortical neurons had little effect on the axonal contralateral branching/targeting (Fig. 2*H*,*I*). However, DCC KD impaired axonal contralateral growth and branching (Fig. 2*H*,*J*). These results, which are in line with our hypothesis, suggest that DCC and Myo X in neurons are necessary to promote axon branching, but Netrin-1, an extracellular cue, regulates axon development in cell-nonautonomous fashion.

### Netrin-1 increasing axonal distribution and transport of Myo X in cultured neurons

**Figure 3.**
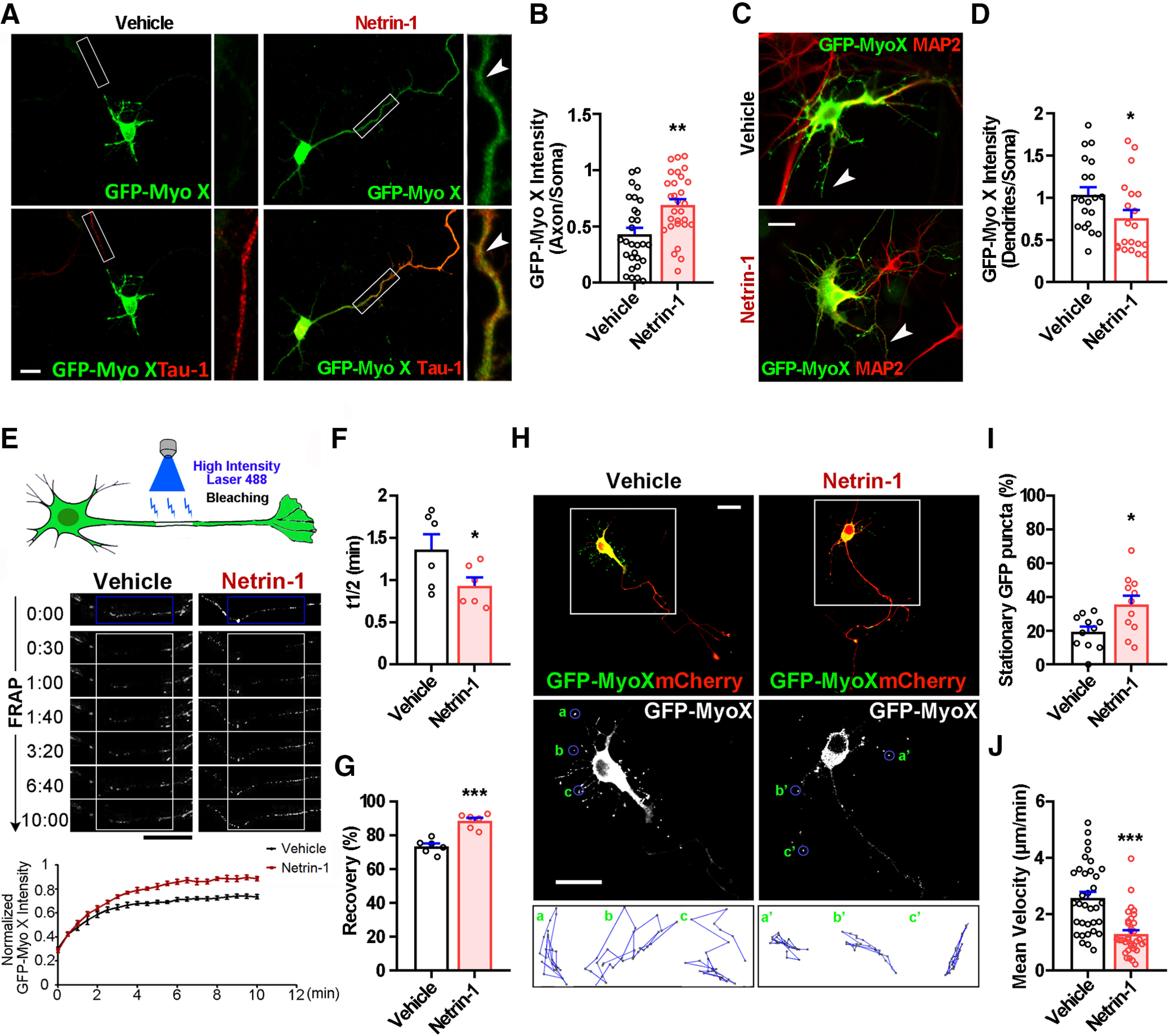
Increase of axonal distribution and anterograde transport of GFP-Myo X by Netrin-1. ***A–D***, Immunostaining analysis using indicated antibodies at DIV3 neurons that were transfected with GFP-Myo X at DIV1 and treated with vehicle or Netrin-1 for 1 h. ***A***, Images marked by rectangles were amplified and shown in the right panels. White arrowheads indicate the axonal distribution of GFP-Myo X. Scale bar, 10 μm. ***B***, Quantification of GFP-Myo X intensity in axons. The axonal GFP-Myo X level was normalized to somatic GFP-Myo X. Student's *t* test, *p* = 0.0012. ***C***, Yellow arrows indicate GFP-Myo X distribution in MAP2-positive dendrites. Scale bar, 10 μm. ***D***, Quantification of GFP-Myo X intensity in dendrites. The dendritic GFP-Myo X level was normalized to somatic GFP-Myo X. Student's *t* test, *p* = 0.0471. ***E–G***, Analysis of axonal GFP-Myo X mobility with FRAP assay. ***E***, Images of FRAP analysis in GFP-Myo X-expressing neurons in the presence of vehicle or Netrin-1 and the quantification (normalized GFP-Myo X intensity of the photobleached axon compartment). Scale bar, 20 μm. ***F***, Quantification of half-time of maximum recovery (t1/2). Student's *t* test, *p* = 0.0446, *n* = 6 neurons from three different experiments. ***G***, Percentage of GFP-Myo X recovery. Student's *t* test, *p* < 0.0001, *n* = 6 neurons from three different experiments. ***H–J***, Time-lapse imaging analysis of GFP-Myo X-expressing neurons in the presence of vehicle or Netrin-1. ***H***, the mCherry was cotransfected to visualize neuronal processes. Images marked in rectangular were amplified and showed in the middle panels. Mobile trajectory of indicated GFP-Myo X puncta was presented in the bottom panels. Scale bar, 20 μm. ***I***, Quantification of mean velocity of GFP-Myo X puncta. Student's *t* test, *p* < 0.0001. ***J***, Quantification of stationary GFP-Myo X. Student's *t* test, *p* = 0.0146. Data are presented as the mean ± SEM. The numbers of neurons scored are from 3 different experiments for each group and indicated on the graphs. *p < 0.05; **p < 0.01; ***p < 0.001.

To understand how Netrin-1 regulates the function of Myo X in axon development, we examined the effect of Netrin-1 on exogenous Myo X (GFP-Myo X) distribution in cultured neurons. To our surprise, GFP-Myo X was largely distributed in the soma and the tips of dendrite-like filopodia, but was low in Tau-1-positive axonal compartments in the absence of Netrin-1 (Fig. 3*A*). Upon Netrin-1 stimulation, an obvious increase of GFP-Myo X in Tau-1-positive axons with a slight decrease of GFP-Myo X in MAP2-positive dendritic neurites were detected (Fig. 3*A–D*), suggesting a role of Netrin-1 in regulating GFP-Myo X distribution in axons and dendrites.

Next, we examined the dynamics of GFP-Myo X in axons by FRAP (Fig. 3*E*). At 3 d *in vitro* (DIV3), neurons exhibited a type of polarized morphology with a very long axon and multiple short dendrites. The longest neurite, likely to be the axon (Dotti et al., 1988), was analyzed. The fluorescence recovery of GFP-Myo X in control axons was much slower and more incomplete than that of Netrin-1-treated axons (Fig. 3*E–G*), supporting the view for Netrin-1 to enhance GFP-Myo X movement. In contrast to axonal GFP-Myo X, GFP-Myo X in dendrite-like filopodia exhibited a puncta pattern (Fig. 3*H*). Time-lapse imaging and analyzing the motility of GFP-Myo X in these filopodia showed both extension and retraction movements of GFP-Myo X puncta in control neurons (Fig. 3*H*, bottom panels). Upon Netrin-1 stimulation, the traveling path and the average velocity of GFP-Myo X puncta were all reduced (Fig. 3*H*, bottom panels, *I*), with an increase in the percentage of stationary puncta (Fig. 3*J*). Since Myo X motility is coupled with the filopodia movement and MyoX undergoes forward and reverse movement along filopodia (Berg and Cheney, 2002), it is possible that Netrin-1 suppressed both motilities of GFP-MyoX and filopodia. Together, these results suggest that while Netrin-1 increases anterograde movement of GFP-Myo X in axons, it decreases the motility of GFP-Myo X in dendrite-like filopodia.

### Requirement of DCC and PI3K activation for Netrin-1 increased axonal distribution of Myo X

**Figure 4.**
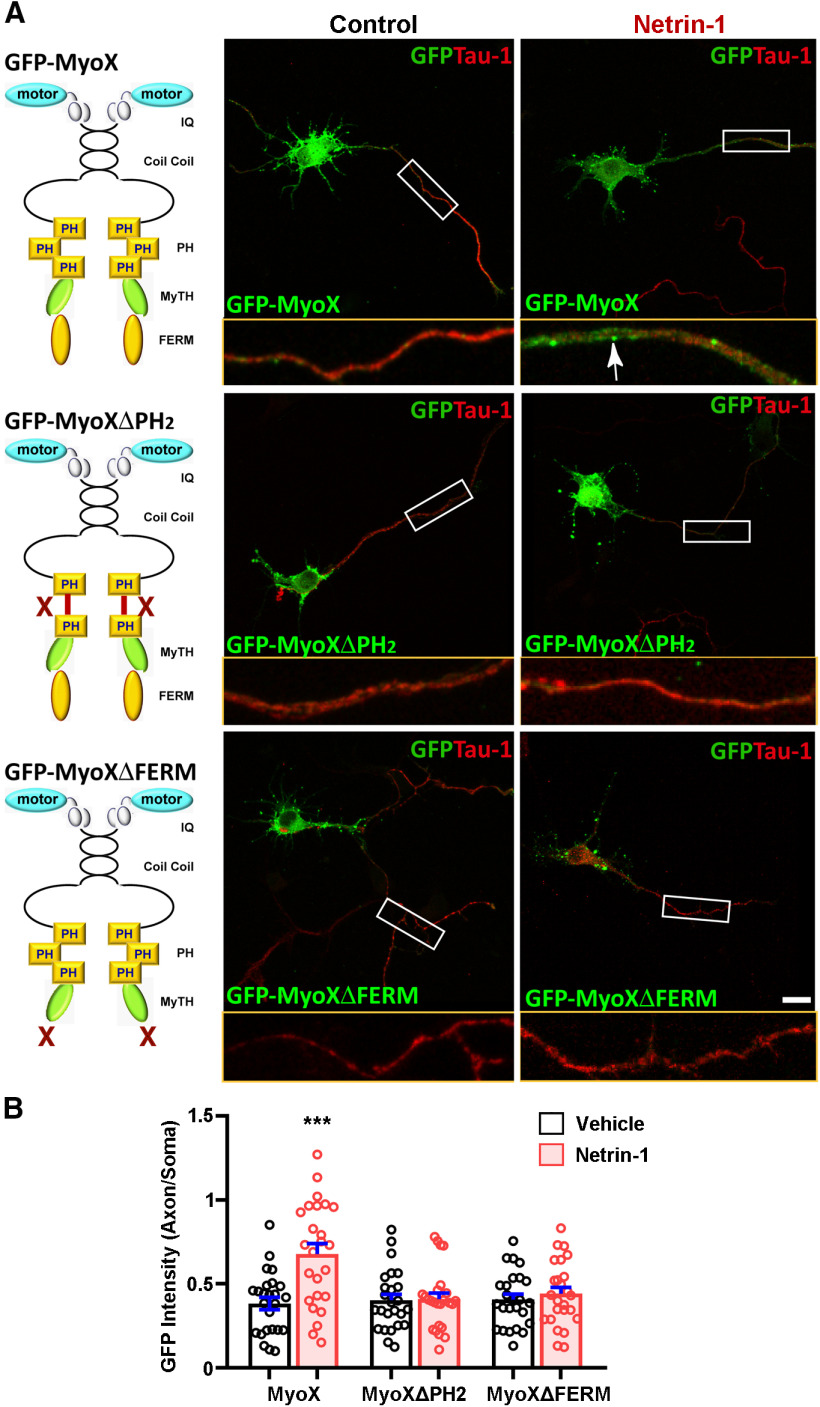
Requirement of the PH and FERM domains of Myo X for Netrin-1 increase of axonal distribution of exogenous GFP-Myo X. ***A***, Neurons transfected with GFP-Myo X and its deletion mutants (illustrated in the left panels) were treated with vehicle or Netrin-1 for 1 h and then stained with anti-Tau-1 antibody at DIV3. Scale bar, 10 μm. ***B***, Quantification of axonal distribution of GFP-Myo X or its deletion mutants. Student's *t* test: for Myo X group, *p* = 0.0003; for Myo Δ PH group, *p* = 0.83; for Myo Δ FERM group, *p* = 0.46. Data are presented as the mean ± SEM. The numbers of neurons scored in these groups are from three different experiments and are indicated on the graphs. ns, No significant difference; ***p < 0.001.

To further understand how Netrin-1 regulates the axonal distribution of Myo X, we first mapped domains in GFP-Myo X that are necessary for this event. Myo X is a multidomain containing unconventional myosin, containing 3 PH domains, an MyTh4 domain, and a band in the FERM domain in the C terminus, in addition to the motor domain in the N terminus (Berg et al., 2000; Kerber and Cheney, 2011). GFP-Myo X deletion mutants were generated as illustrated in Figure 4*A* (left). In the absence of Netrin-1, Myo X mutants containing the motor domain showed a distribution pattern similar to that of full-length Myo X, with predominant localizations in the soma and dendritic filopodia (Fig. 4*A*,*B*). However, in the presence of Netrin-1, Myo X deletions in the second PH domain (Myo X^ΔPH2^) or in the FERM domain (Myo X^ΔFERM^) abolished Netrin-1-induced axonal distribution (Fig. 4*A*,*B*). These results suggest a requirement of both PH and FERM domains in Myo X for Netrin-1-induced axonal distribution of Myo X.

**Figure 5.**
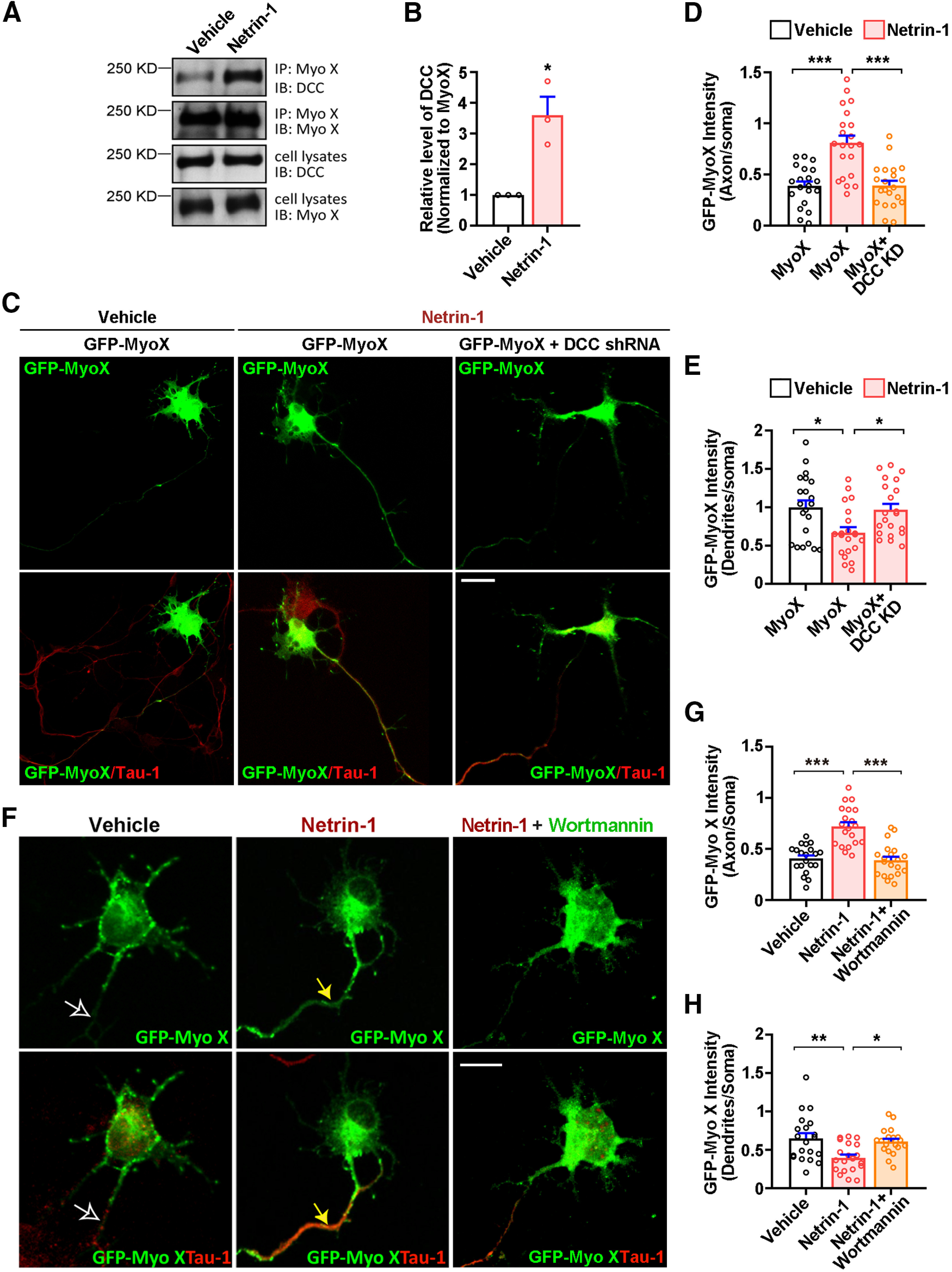
DCC and PI3K activity-dependent axonal distribution of GFP-Myo X in response to Netrin-1 stimulation. ***A***, Immunoprecipitation analysis of DCC association with Myo X in cultured cortical neurons that were treated with vehicle or Netrin-1 for 1 h. ***B***, Quantifications of DCC binding with MyoX. Student's *t* test, *p* = 0.0122. ***C***, Neurons transfected with GFP-Myo X or GFP-Myo X together with DCC miRNA were treated with vehicle or Netrin-1 and then stained with anti-Tau-1 antibody at DIV3. Scale bar, 10 μm. ***D***, Quantification of axonal distribution of GFP-Myo X. One-way ANOVA, for Myo X group with vehicle stimulation and Myo X group with Netrin-1 stimulation, *p* < 0.0001; for Myo X and Myo X+DCC-KD groups, *p* < 0.0001. ***E***, Quantification of dendritic distribution of GFP-Myo X. One-way ANOVA, for Myo X groups with vehicle and Myo X group with Netrin-1 stimulation, *p* = 0.0142, for Myo X and Myo X+DCC-KD groups, *p* = 0.0283. ***F***, GFP-Myo X-expressing neurons were treated with vehicle and Netrin-1 with or without PI3 kinase inhibitor, wortmannin (10 nm) for 1 h and subjected to immunostaining with anti-Tau-1 antibody at DIV3. Scale bar, 10 μm. ***G***, Quantification of axonal distribution of GFP-Myo X. One-way ANOVA, *p* < 0.0001 for both groups. ***H***, Quantification of dendritic distribution of GFP-Myo X. One-way ANOVA, for vehicle and Nerin-1 groups, *p* = 0.0023; for Netrin-1 and Netrin-1+wortmannin groups, *p* = 0.0124. Data are presented as the mean ± SEM. The numbers of brain sections scored are from three different brains for each group and indicated on the graphs. ns, No significant difference; *p < 0.05; **p < 0.01; ***p < 0.001.

As the FERM domain in Myo X binds to DCC (Zhu et al., 2007), we thus speculate that DCC–Myo X interaction may be critical for this event. Indeed, this view is supported by the observations that DCC–Myo X interaction is upregulated by Netrin-1 (Fig. 5*A*,*B*), and neurons suppressing DCC expression by its shRNA failed to response to Netrin-1 in targeting Myo X to axons (Fig. 5*C–E*).

PH domains of Myo X are known to bind to multiphosphoinositols, including PI(4,5)P2, PI(3,4)P2, and PI(3,4,5)P3 (Cox et al., 2002; Umeki et al., 2011). The second PH domain is crucial for binding to PI(3,4,5)P3/PI(3,4)P2, products of PI3K activation (Umeki et al., 2011). As this PH domain in Myo X is necessary for Netrin-1-induced axonal distribution of Myo X, we wondered whether PI(3,4,5)P3/PI(3,4)P2 are involved in the axonal distribution of Myo X. To this end, neurons expressing GFP-Myo X were treated with Netrin-1 in the presence or absence of wortmannin (10 nm), an inhibitor of PI3K that blocks the production of PI(3,4,5)P3 and PI(3,4)P2. Such an inhibition abolished Netrin-1-induced axonal distribution of Myo X (Fig. 5*F–H*), supporting a role for PI3K activation and its products, PI(3,4,5)P3/PI(3,4)P2, in this event.

### Myo X interaction with KIF13B, a kinesin family motor protein

**Figure 6.**
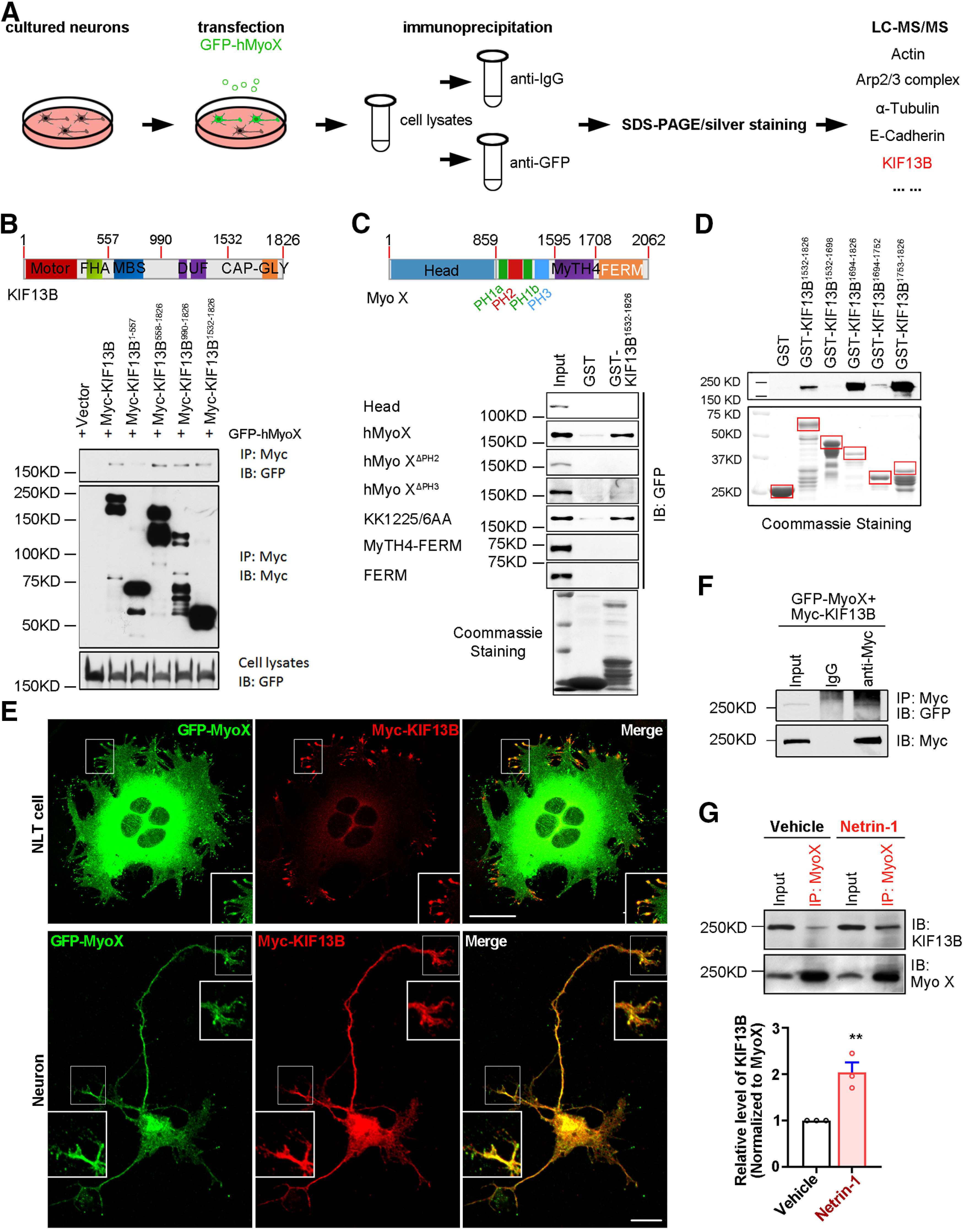
Interaction of Myo X with KIF13B. ***A***, KIF13B was identified as a binding partner of MyoX by LC-MS/MS. ***B***, Coimmunoprecipitation of GFP-hMyo X and Myc-KIF13B^1532-1826^. GFP-hMyo X was coexpressed with Myc-KIF13B and its deletion mutants in HEK293T cells and immunoprecipitated by anti-Myc antibody. ***C***, Immunoblotting of the pulled down fraction by the GST-KIF13B C-terminus fusion protein and GST alone. The numbers of GST fusion proteins and GST were revealed by Coomassie blue staining (bottom). ***D***, Immunoblotting of the pulled down fraction by the truncated C-terminal domains fused with GST and GST alone. The amounts of different GST fusion proteins and GST alone were revealed by Coomassie blue staining (bottom). ***E***, Colocalization of GFP-Myo X and Myc-KIF13B in filopodia tips of NLT cells (top panels) and in axon and dendrite-like filaments in cultured cortical neurons (bottom panels). Images marked in rectangular was amplified and included as insert. Scale bar, 20 μm. ***F***, Coimmunoprecipitation of exogenous Myc-KIF13B and GFP-Myo X. HEK293T cell lysates were immunoprecipitated with anti-Myc antibody and with IgG as control. ***G***, Immunoprecipitation of endogenous Myo X and KIF13B with or without Netrin-1 stimulation. Neurons treated with vehicle or Netrin-1 were lysed and incubated with anti-Myo X antibody (top). Quantifications of KIF13B binding with MyoX are presented in the bottom panel. Student's *t* test, *p* = 0.0089. Data are presented as the mean ± SEM. For statistical analysis, three independent experiments were performed and indicated on the graphs. **p < 0.01.

To further investigate the underlying mechanisms of Netrin-1-stimulated Myo X anterograde transport, we attempted to identify additional Myo X binding proteins by use of the LC-MS/MS to sequence the proteins associated with the Myo X immunoprecipitates. Specifically, GFP-hMyoX (GFP-tagged headless MyoX) was transfected into cultured cortical neurons. The cell lysates were immunoprecipitated with anti-IgG (as a control) or anti-GFP antibody. The immune complexes were resolved by SDS-PAGE and subjected to silver staining. All specific bands were subjected to LC-MS/MS. Five hundred eighty-six proteins were detected in the immunoprecipitates with anti-GFP antibody, including previously reported binding partners, such as actin, α-tubulin, Arp2/3 complex, and E-cadherin (Fig. 6*A*). Interestingly, KIF13B (polypeptide amino acid sequence, LSLVDLAGSER, TVAATNMNEESSR, GSLLSEPAIQVR, DVPTGGIFQLR; accession #E9Q4K7) was identified (Fig. 6*A*). Given that anterograde transport is powered by kinesin family motor (Hirokawa et al., 2010), we thus asked whether Myo X acted as a cargo of KIF13B to be transported along microtubules in axons. It is of interest to note the report that KIF13B is essential for anterograde transport of PI(3,4,5)P3 for axonal outgrowth and formation (Yoshimura et al., 2010). We then mapped the domains in KIF13B for its interaction with Myo X by coimmunoprecipitation assay. KIF13B contains a motor domain at the NH2 terminus, a FHA (forkhead-associated) domain, a MAGUK binding stalk domain, two domains of unknown function and a CAP-Gly motif at the C terminus (Fig. 6*B*). As shown in Figure 6, *B* and *C*, terminal regions of KIF13B (Myc tagged; including KIF13B^558-1826^, KIF13B^990-1826^, and KIF13B^1532-1826^), but not the N terminus (KIF13B^1-557^), were detected in Myo X immunoprecipitates. Further analysis of their interaction identified that the C-terminal domain KIF13B^1532-1826^ is involved in the interaction with Myo X.

The Myo X-KIF13B interaction was further verified by a GST pulldown assay. The recombinant GST-KIF13B^1532-1826^ fusion protein was produced (Fig. 6*C*), which was used to pull down lysates expressing various GFP-Myo X mutants (including Myo X^Head^, hMyo X, hMyo X^ΔPH2^, hMyo X^ΔPH3^, hMyo X^KK1225/6AA^, Myo X^Myth4-Ferm^, and Myo X^Ferm^). hMyoX is an abbreviation of headless MyoX, which contains amino acids from 860 to 2062. hMyoX^KK1225/6AA^ means that the 1225/1226 lysine was further mutated to alanine. These two lysines are located in the second PH domain and are required for Myo X binding with PI(3,4,5)P3. Note that only hMyo X and hMyo X^KK1225/6AA^ were pulled down by GST-KIF13B^1532-1826^, suggesting the requirement of the second and third PH domains of Myo X for its binding to the C-terminal domain in KIF13B (Fig. 6*C*). By this assay, the effective Myo X binding region in KIF13B was further mapped to the last 74 aa in its C terminus (Fig. 6*D*). It is noteworthy that while the site KK1225/6 in Myo X is critical for binding to PI(3,4,5)P3 (Plantard et al., 2010), it was not required for Myo X interaction with KIF13B.

Finally, we examined Myo X–KIF13B interaction by coimmunostaining analysis. As shown in Figure 6*E*, GFP-Myo X was colocalized with Myc-KIF13B in filopodia tips in NLT cells and in axon and dendrite-like filaments in cultured cortical neurons. In addition, their interaction was reconfirmed by coimmunoprecipitation analysis of exogenously expressed GFP-Myo X and Myc-KIF13B (Fig. 6*F*), as well as endogenous KIF13B with Myo X in primary neuronal lysates (Fig. 6*G*). Interestingly, Netrin-1 stimulation increased Myo X–KIF13B interaction in neurons (Fig. 6*G*). Together, these results suggest that Myo X interacts with KIF13B, implicating KIF13B in Netrin-1-induced Myo X distribution in axons.

### Myo X as a cargo of KIF13B for its axonal distribution

**Figure 7.**
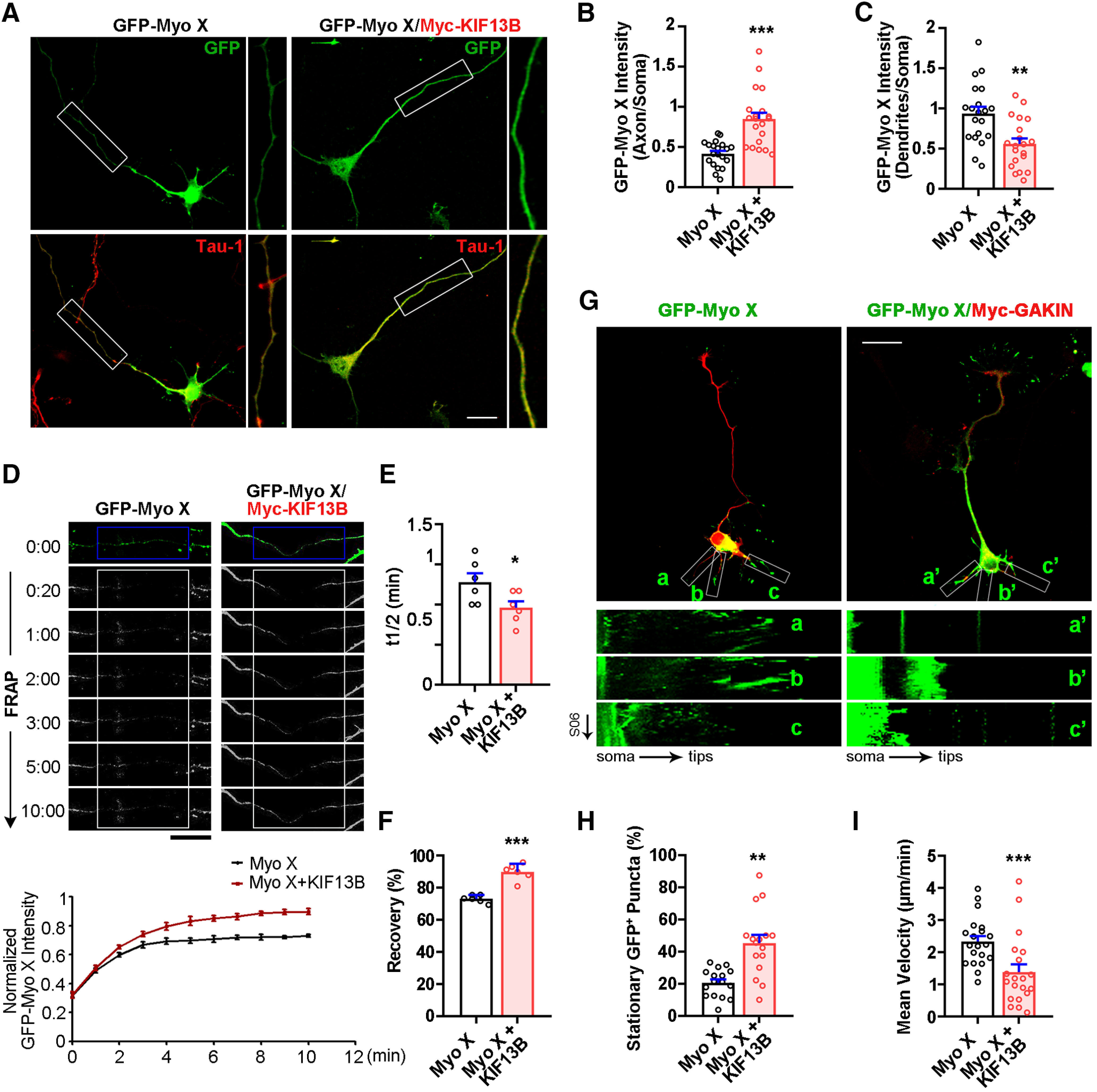
Involvement of KIF13B in Myo X anterograde transportation. ***A–C***, Immunostaining analysis using indicated antibodies at DIV3 neurons that were transfected with GFP-Myo X or GFP-Myo X together with Myc-KIF13B at DIV1. ***A***, Images marked in a rectangle were amplified and are shown in the right panels. Scale bar, 20 μm. ***B***, Quantification of GFP-Myo X intensity in axons. The axonal GFP-Myo X level was normalized to somatic GFP-Myo X. Student's *t* test, *p* < 0.0001. ***C***, Quantification of GFP-Myo X intensity in dendrites. The dendritic GFP-Myo X level was normalized to somatic GFP-Myo X. Student's *t* test, *p* = 0.0013. ***D–F***, Analysis of KIF13B effect on axonal GFP-Myo X mobility with FRAP assay. ***D***, Images of FRAP analysis in DIV3 cortical neurons that were transfected with GFP-Myo X or GFP-Myo X together with Myc-KIF13B at DIV1 and the quantification (normalized GFP-Myo X intensity of the photobleached axon compartment). Scale bar, 20 μm. ***E***, Quantification of half-time of maximum recovery. Student's *t* test, *p* = 0.0213, *n* = 6 neurons from three different experiments. ***F***, Percentage of GFP-Myo X recovery. Student's *t* test, *p* < 0.0001, *n* = 6 neurons from three different experiments. ***G–I***, Time-lapse imaging analysis of GFP-Myo X together with vector or GFP-Myo X together with Myc-KIF13B-transfected neurons. ***G***, The marked rectangle in ***G*** were further analyzed by kymographs (see bottom panels), which show the mobility of GFP-Myo X-positive vesicles during 5 min recordings. Vertical lines represent stationary Myo X-vesicles; oblique lines or curves to the right represent anterograde movements, and lines to the left indicate retrograde transport. Scale bar, 20 μm. ***H***, Quantification of the mean velocity of GFP-Myo X puncta. Student's *t* test, *p* = 0.0026. ***I***, Quantification of stationary GFP-Myo X. Student's *t* test, *p* = 0.0001. Data are presented as the mean ± SEM. The numbers of cells scored are from three different brains for each group and are indicated on the graphs. *p < 0.05; **p < 0.01; ***p < 0.001.

To investigate the function of KIF13B in Netrin-1-induced Myo X anterograde transportation, we first examined whether the distribution of GFP-Myo X in Tau-1-positive axons was affected by KIF13B expression. Indeed, the expression of KIF13B was sufficient to increase the localization of GFP-Myo X in axons and decrease the localization of GFP-Myo X in dendrites in the absence of Netrin-1(Fig. 7*A–C*). In line with this view was the observation by the FRAP assay that the recovery of GFP-Myo X after photobleaching in axons was sped up by KIF13B expression (Fig. 7*D–F*). Furthermore, the effect of KIF13B on GFP-Myo X distribution was examined by time-lapse imaging analysis. As shown in Figure 7*G*, GFP-Myo X puncta exhibited high motility in dendrite-like neurites. Such actin-based motility of GFP-Myo X was decreased in neurons coexpressing KIF13B (Fig. 7*G–I*).

**Figure 8.**
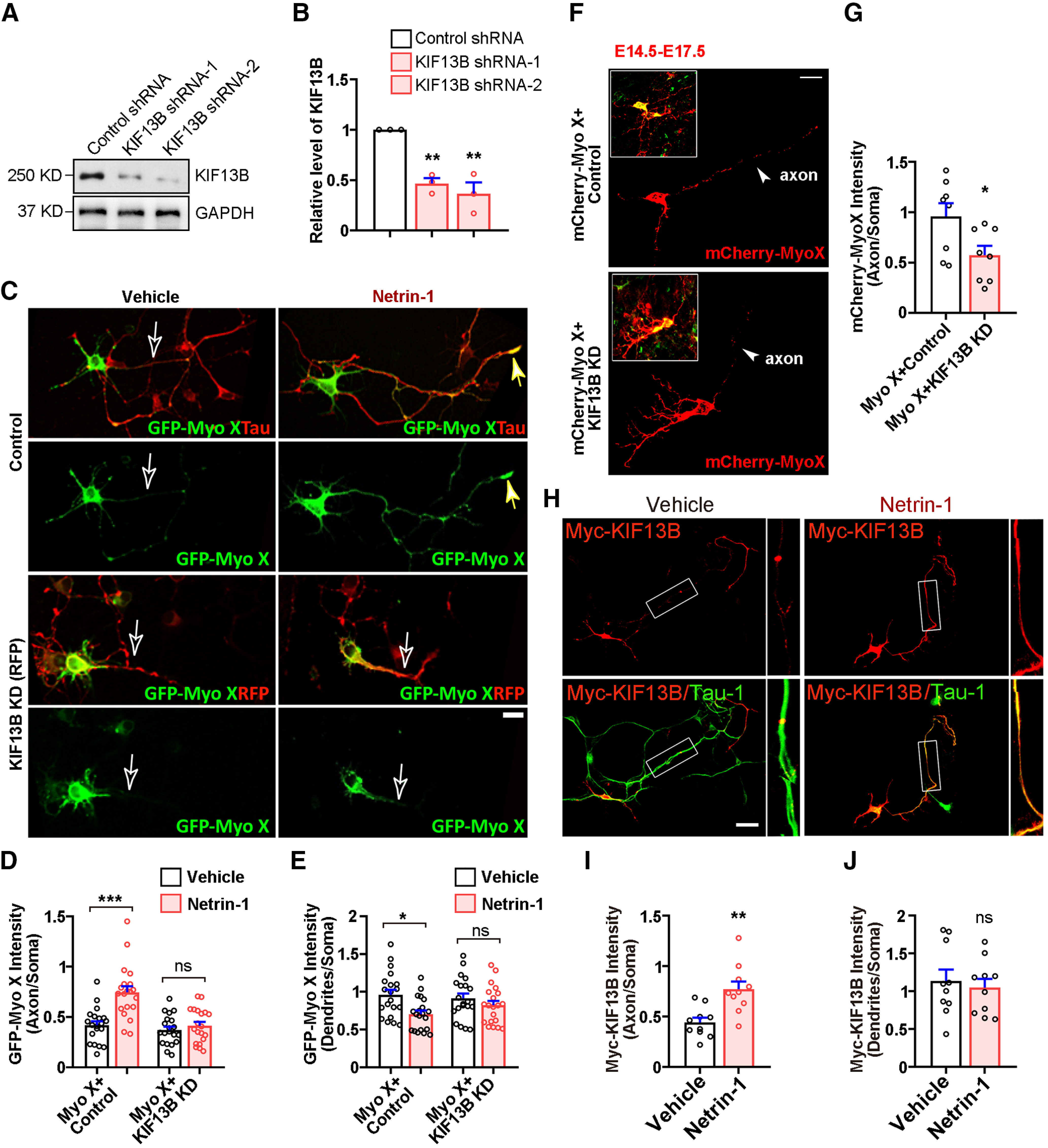
Requirement of KIF13B for Netrin-1 induced axonal distribution of GFP-Myo X. ***A***, Western blot showing the silence effect of KIF13B shRNA in cultured cortical neurons. ***B***, Quantification of KIF13B knock-down efficiency. One-way ANOVA, *p* = 0.0036 for KIF13B shRNA-1 group; *p* = 0.0015 for KIF13B shRNA-2 group. ***C***, Neurons transfected with GFP-Myo X together with control shRNA or KIF13B shRNA, respectively, were treated with vehicle or Netrin-1 for 1 h and subjected to immunostaining at DIV3. Scale bar, 10 μm. ***D***, Quantification of GFP-Myo X intensity in axons. The axonal GFP-Myo X level was normalized to somatic GFP-Myo X. One-way ANOVA: *p* < 0.0001 between GFP-Myo X+Control groups; *p* = 0.91 between GFP-Myo X+KIF13B-KD groups. ***E***, Quantification of GFP-Myo X intensity in dendrites. The dendritic GFP-Myo X level was normalized to somatic GFP-Myo X. One-way ANOVA: *p* = 0.0293 between GFP-Myo X+Control groups; *p* = 0.88 between GFP-Myo X+KIF13B-KD groups. ***F***, Representative images of cortical neurons electroporated with indicated plasmids at E15.5. Scale bar, 10 μm. ***G***, Quantification of mCherry-Myo X intensity in axons. The axonal mCherry-Myo X level was normalized to somatic mCherry-Myo X. Student's *t* test, *p* = 0.0331. ***H***, Neurons transfected with Myc-KIF13B were treated with vehicle or Netrin-1 for 1 h and subjected to immunostaining with indicated antibodies at DIV3. Images marked in rectangles were amplified and are shown in the right panels. Scale bar, 20 μm. ***I***, Quantification of Myc-KIF13B intensity in axons. The axonal Myc-KIF13B level was normalized to somatic Myc-KIF13B. Student's *t* test, *p* = 0.0026. ***J***, Quantification of Myc-KIF13B intensity in dendrites. The dendritic Myc-KIF13B level was normalized to somatic Myc-KIF13B. Student's *t* test, *p* = 0.59. Data are presented as the mean ± SEM. The numbers of neurons scored in these groups are from three different experiments and indicated on the graphs. ns, No significant difference; *p < 0.05; **p < 0.01; ***p < 0.001.

Second, we determined whether KIF13B was necessary for Netrin-1-induced Myo X axonal distribution. A plasmid encoding KIF13B shRNA was generated, which selectively suppressed KIF13B expression (Fig. 8*A*,*B*). GFP-Myo X with KIF13B shRNA or control shRNA were cotransfected into neurons treated with or without Netrin-1. In control neurons (GFP-Myo X with control shRNA), the distribution of GFP-Myo X in axons was increased and in dendrites was decreased by Netrin-1 stimulation (Fig. 8*C–E*). However, such an increase of the axonal distribution of Myo X was abolished in neurons cotransfected with KIF13B shRNA (Fig. 8*C*,*D*). Moreover, KIF13B KD in embryonic mouse cortical neurons significantly suppressed GFP-Myo X distribution in axon-like neurites (Fig. 8*F*,*G*). Together, these results suggest that KIF13B is not only sufficient, but also necessary for Netrin-1-induced Myo X distribution in axons. In line with this view, the distribution of KIF13B in axons was increased by Netrin-1 (Fig. 8*H–J*).

### KIF13B, as Myo X, promoting axonal initiation and contralateral branching/targeting in developing cerebral cortex

**Figure 9.**
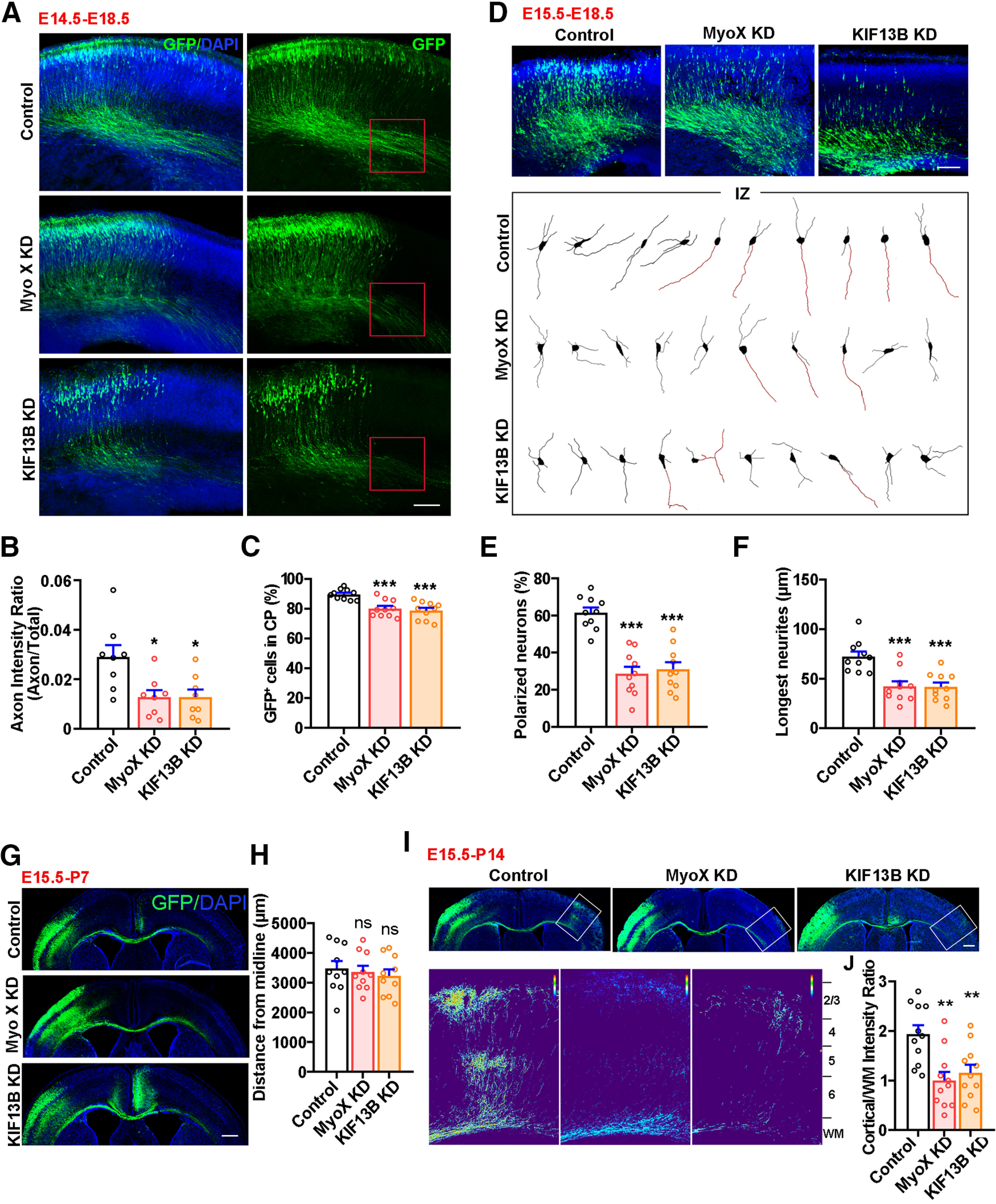
Similar roles of KIF13B in axon initiation and branching as Myo X. ***A***, Representative images of E18.5 cerebral cortex electroporated with control shRNA (Control), KIF13B shRNA (KIF13B-KD), and Myo X miRNA (Myo X-KD) plasmids at E14.5. Scale bar, 100 μm. ***B***, Quantification of axon initiation by using the axon intensity ratio. Student's *t* test: for Myo X-KD group, *p* = 0.0150; for KIF13B-KD group, *p* = 0.0102. ***C***, Quantification of GFP-positive cells in CP. Student's *t* test: for Myo X-KD group, *p* = 0.0002; for KIF13B-KD group, *p* < 0.0001. ***D***, Neurons in the IZ of E18.5 cerebral cortex in each group. Mouse cortices electroporated with indicated plasmids were presented in the top panels. The tracings of representative GFP-positive neurons in each group are presented in the bottom panels. The longest neurite (with length >50 μm and two times more than that of the second longest one) is considered as axon and is marked in red. Scale bar, 100 μm. ***E***, Quantification of polarized neurons in IZ. Student's *t* test: for Myo X-KD group, *p* < 0.0001; for KIF13B-KD group, *p* < 0.0001. ***F***, Quantification of the length of longest neurites. Student's *t* test: for Myo X-KD group, *p* = 0.0003; for KIF13B-KD group, *p* = 0.0003. ***G***, ***H***, Representative images of P7 cerebral cortex electroporated with above indicated plasmids at E15.5 as well as quantification of axon elongation. Scale bar, 500 μm. Student's *t* test: for Myo X-KD group, *p* = 0.9059; for KIF13B-KD group, *p* = 0.65. ***I***, ***J***, Representative images of P14 cerebral cortex electroporated with above indicated plasmids at E15.5 as well as quantification of axon contralateral branching. Scale bar, 100 μm. Student's *t* test: for Myo X-KD group, *p* = 0.0013; for KIF13B-KD group, *p* = 0.0067. Data are presented as the mean ± SEM. The numbers of brain sections or cells scored are from three different brains for each group and indicated on the graphs. ns, No significant difference; *p < 0.05; **p < 0.01; ***p < 0.001.

The important role of KIF13B in Netrin-1-induced Myo X axonal distribution led us to speculate that that KIF13B plays a role similar to that of Myo X in Netrin-1-induced axonal initiation and targeting *in vivo*. To test this speculation, KIF13B shRNA and Myo X miRNA were transported by IUE into the progenitor cells of cortical neurons in E14.5 mouse embryos, and their brain sections at E18.5 were examined (Fig. 9*A*). As shown in Figure 9, *A* and *B*, KIF13B KD resulted in a decrease in axon intensity ratio in the cortical brains, an impairment in axon initiation similar to that of Myo X-KD neurons. In addition, the percentage of Myo X- or KIF13B-deficient neurons into CP was decreased compared with that of control neurons (Fig. 9*A*,*C*). Furthermore, we analyzed the polarization of neurons in IZ and defined the longest neurites as axons by the following two criteria: the length of the longest neurite is >50 μm and is two times more than that of the second longest one. Based on these criteria, the percentages of polarized neurons in both Myo X-KD and KIF13B-KD groups were decreased (Fig. 9*D*,*E*) and their axons were also shorter (Fig. 9*D*,*F*). These results suggest that KIF13B plays a role similar to that of Myo X in axon initiation.

We next determined whether KIF13B regulates axon projection and branching as Myo X does. To this end, KIF13B was suppressed in E15.5 embryos by IUE of its shRNA (GFP). At neonatal age (e.g., P7 and P14), the neurons electroporated mostly migrated into cortical L2/3 pyramidal neurons whose axons project to the contralateral side via CC (Alcamo et al., 2008). Just as with Myo X-KD axons, axons of KIF13B-KD neurons crossed the midline at P7, without an obvious reduction in their axonal length (Fig. 9*G*,*H*). However, at P14, the axonal contralateral branches were severely diminished in KIF13B-KD neurons compared with those of controls (Fig. 9*I*,*J*). Together, these results suggest that KIF13B is necessary to promote axon initiation and branching/targeting in developing cortical neurons, providing additional support for KIF13B as an important mediator for Netrin-1-induced and Myo X-regulated axon initiation and targeting.

## Discussion

In this study, we present evidence that Netrin-1 increases axonal targeting of Myo X in neurons. This event is essential for axon initiation and contralateral branching, but not midline crossing. Further mechanistic studies suggest that Netrin-1 increases Myo X interaction with KIF13B, thus promoting axonal transport of Myo X, axonal initiation, and branching/targeting. These results reveal a new mechanism underlying Netrin-1-regulated axon pathfinding.

As an unconventional Myosin family protein, Myo X is widely expressed and implicated in multiple cellular functions in different cell types, including Netrin-1-induced neurite outgrowth and growth cone guidance; Zhu et al., 2007), BMP6-dependent filopodial migration and activation of BMP receptors (Pi et al., 2007), and migration of *Xenopus* cranial neural crest cells (Hwang et al., 2009; Nie et al., 2009). While it is evident that Myo X modulates growth cone actin dynamics and promotes axon specification in cultured neurons (Yu et al., 2012), the *in vivo* evidence for this view is limited. Here, we found that Myo X is critical for axon initiation and terminal branching/targeting in the developing neocortex. Myo X KD (by RNA interference) or KO (by Cre-Loxp combination) decreased the axon intensity ratio, suggesting a deficit in axon initiation (Figs. 1*E*, 9*A*). Morphology analysis of individual neurons suggested an impairment of axon genesis (Fig. 9*D–F*). In addition to axon initiation, Myo X KD or KO also impaired axon terminal branching/targeting (Fig. 1*C*,*I*). This function of Myo X is in line with the observations that Myo X plays an important role in regulating axon filaments and actin filaments in the leading margin of growth cones, which are responsible for perceiving extrinsic guidance factors or adhesive signals and producing traction for axon terminal elongation toward its target (Dent and Gertler, 2003; Yu et al., 2012). Interestingly, Netrin-1 KD in cortical neurons had little effect on the axonal contralateral branching/targeting, while DCC KD impaired axonal contralateral branching/targeting (Fig. 2*F*,*G*). These results together suggest that DCC and Myo X in neurons play a cell-autonomous role in promoting axon branching, while Netrin-1, as an extracellular cue, regulates axon development in a nonautonomous way. Although Netrin-1-DCC-Myo X was involved in axon branching, it is not the only pathway underlying axon branching. It is of interest to note that Netrin-1 promotes exocytosis and plasma membrane expansion for axon branching via TRIM9 release of SNAP25 and SNARE-mediated vesicle fusion (Winkle et al., 2014). It will be of interest to investigate whether Myo X is involved in Netrin-1-stimulated exocytosis for axon branching. Notice that Myo X KO or KD has little effect on axon midline crossing, as it does in Netrin-1-KO or DCC-KD axons (Fig. 2*F*,*G*). These results suggest a neuronal DCC-Myo X-independent mechanism for axon midline crossing.

Axons contain abundant microtubules, although their growth cones have enriched actin filaments (Dent and Gertler, 2003). How is Myo X, an actin filament-based motor protein, transported to the growth cones of axons? Although Myo X interacts with microtubules with its MyTH4 domain (Weber et al., 2004; Woolner et al., 2008; Wühr et al., 2008), little evidence demonstrates that Myo X has microtubule-based motor activity. Thus, we speculate that the microtubule-dependent motor protein kinesin may be responsible for Myo X anterograde transportation in axons. To this end, KIF13B was identified as a Myo X binding partner to be responsible for Myo X anterograde transportation. Interestingly, KIF13B, a kinesin family motor protein, plays an essential role in anterograde transport of PI(3,4,5)P3 (Horiguchi et al., 2006), a binding partner and regulator of Myo X (Figs. 6, 7). Moreover, KIF13B exerts functions similar to those of Myo X in promoting axon initiation and terminal targeting (Fig. 9). In aggregates, our results suggest that Myo X appears to be a cargo of KIF13B during its axonal transportation, and at the same time, the actin-based motor activity of Myo X is suppressed by KIF13B.

Netrin-1/DCC signaling is involved in many aspects of axon development, including axon outgrowth and guidance, growth cone steering, and axon branching. The canonical model for the function of Netrin-1 in axon guidance is that Netrin-1 acts as a long-range diffusible guidance cue, centered in the midline (e.g., floor plate in the developing spinal cord), attracting or repulsing axons for their midline crossing. Recent studies have shown that Netrin-1 is produced not only in the midline, but also in neural progenitor cells in the VZ, and is deposited on the pial surface as a haptotactic adhesive substrate, where it guides DCC-positive axon growth (Dominici et al., 2017). In developing cerebral cortex, Netrin-1 mRNA is highly expressed in VZ/SVZ (Zhang et al., 2018). It is of interest to note the report by Brignani et al. (2020) that Netrin-1, produced in the forebrain and provided to the midbrain through axon projections, instructs the migration of GABAergic neurons into the ventral SN, which supports the expression of Netrin-1 in developing neurons in forebrain (Brignani et al., 2020). In line with these reports are observations by Allen's *in situ* and single-cell RNA-seq databases on the websites. These results implicate that Netrin-1/DCC signaling in local microenvironment surrounding newborn neurons is important for axon development. In line with this view, we found that the axon initiation was slowed down by Netrin-1 KO in the local region (Fig. 2*A*,*B*), and Netrin-1 overexpression diminished Myo X deficiency-induced axon initiation deficit. Netrin-1 was knocked out in neural stem cells, as well as their progeny, including neurons and glial cells, which likely reduce Netrin-1 in the local environment. Therefore, the defect in Netrin-1-KO neurons could be because of loss of the autocrine and/or local paracrine effect of Netrin-1. How does Netrin-1 rescue the axon initiation deficit in Myo X-deficient neurons? It is noteworthy that neurons with Myo X KD by its miRNA, unlike Myo X KO, still contained low levels of Myo X. Upon Netrin-1 overexpression, it may promote the axonal transportation of the remaining Myo X and thus diminish the axon initiation defect. Alternatively, Netrin-1 may increase axonal initiation in a Myo X-independent manner, as multiple downstream signals can be activated by Netrin-1.

**Figure 10.**
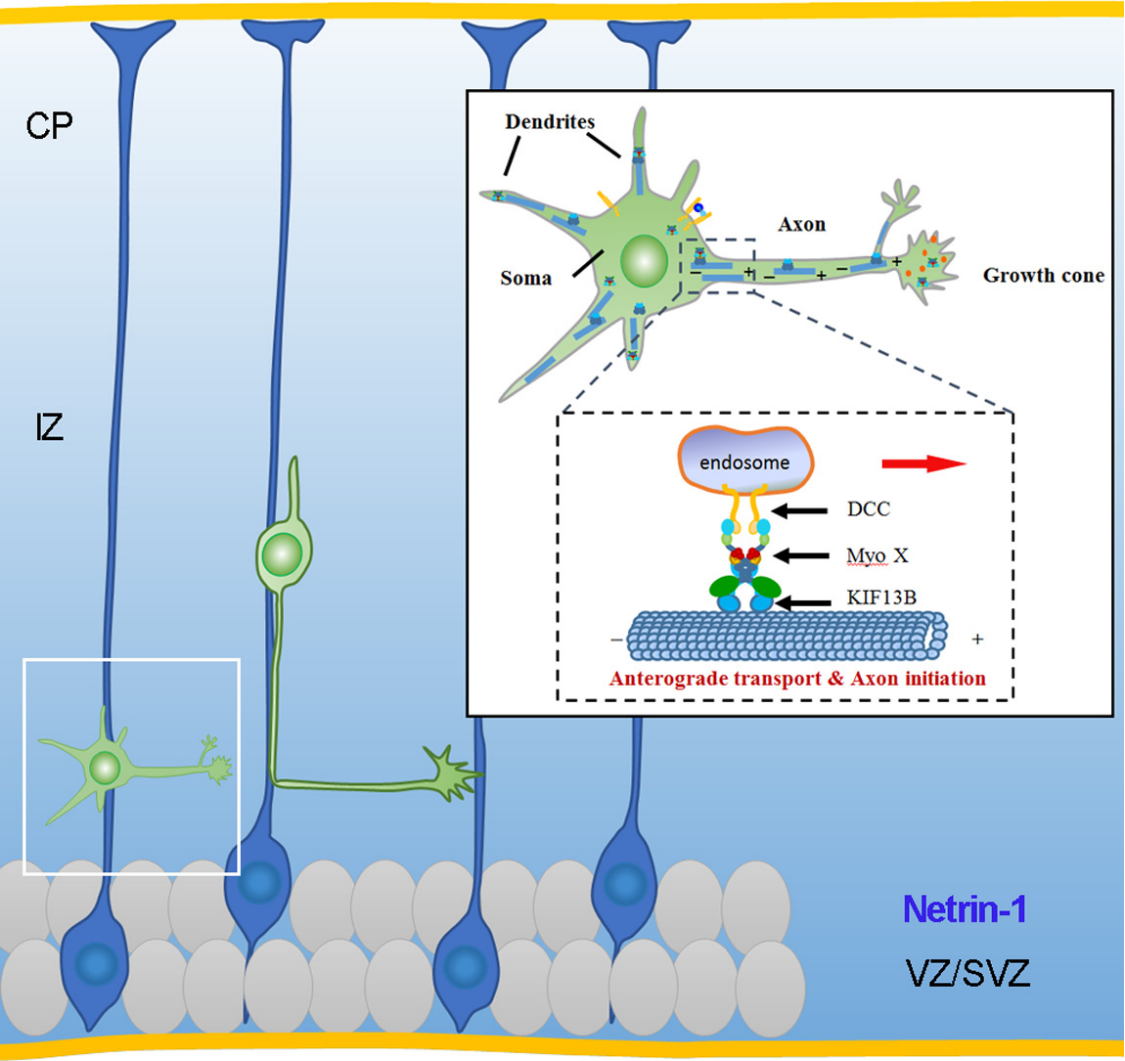
Graphical abstract.

In light of our results, we speculate the existence of the DCC–Myo X–KIF13B complex. Netrin-1 may increase the complex formation by generating more PI(3,4,5)P3, which binds to Myo X, changes Myo X conformation for DCC and KIF13B binding, and then undergoes anterograde transport along microtubules (Fig. 10). The motor activity of Myo X may be suppressed by disconnecting Myo X with F-actin filaments, as we can see that KIF13B suppress Myo X motility in dendrite-like actin filaments. In this complex, Myo X acts as a central adapter protein to link its cargos of DCC and PI(3,4,5)P3/PI(3,4)P2 with KIF13B. Such a Myo X-containing complex may be crucial for Netrin-1-induced axonal outgrowth and growth cone-attractive response.
